# A Study on the Feasibility of the Deep Brain Stimulation (DBS) Electrode Localization Based on Scalp Electric Potential Recordings

**DOI:** 10.3389/fphys.2018.01788

**Published:** 2019-01-04

**Authors:** Maria Ida Iacono, Seyed Reza Atefi, Luca Mainardi, Harrison C. Walker, Leonardo M. Angelone, Giorgio Bonmassar

**Affiliations:** ^1^Athinoula A. Martinos Center for Biomedical Imaging, Department of Radiology, Massachusetts General Hospital, Harvard Medical School, Charlestown, MA, United States; ^2^Division of Biomedical Physics, Office of Science and Engineering Laboratories, Center for Devices and Radiological Health, U.S. Food and Drug Administration, Silver Spring, MD, United States; ^3^Bioengineering Department, Politecnico di Milano, Milan, Italy; ^4^Department of Neurology, University of Alabama at Birmingham, Birmingham, AL, United States; ^5^Division of Movement Disorders, University of Alabama at Birmingham, Birmingham, AL, United States

**Keywords:** electroencephalography (EEG), source localization, DBS placement, surgical navigation, finite difference time domain, computational electromagnetic modeling, forward and inverse problem

## Abstract

Deep Brain Stimulation (DBS) is an effective therapy for patients disabling motor symptoms from Parkinson’s disease, essential tremor, and other motor disorders. Precise, individualized placement of DBS electrodes is a key contributor to clinical outcomes following surgery. Electroencephalography (EEG) is widely used to identify the sources of intracerebral signals from the potential on the scalp. EEG is portable, non-invasive, low-cost, and it could be easily integrated into the intraoperative or ambulatory environment for localization of either the DBS electrode or evoked potentials triggered by stimulation itself. In this work, we studied with numerical simulations the principle of extracting the DBS electrical pulse from the patient’s EEG – which normally constitutes an artifact – and localizing the source of the artifact (i.e., the DBS electrodes) using EEG localization methods. A high-resolution electromagnetic head model was used to simulate the EEG potential at the scalp generated by the DBS pulse artifact. The potential distribution on the scalp was then sampled at the 256 electrode locations of a high-density EEG Net. The electric potential was modeled by a dipole source created by a given pair of active DBS electrodes. The dynamic Statistical Parametric Maps (dSPM) algorithm was used to solve the EEG inverse problem, and it allowed localization of the position of the stimulus dipole in three DBS electrode bipolar configurations with a maximum error of 1.5 cm. To assess the accuracy of the computational model, the results of the simulation were compared with the electric artifact amplitudes over 16 EEG electrodes measured in five patients. EEG artifacts measured in patients confirmed that simulated data are commensurate to patients’ data (0 ± 6.6 μV). While we acknowledge that further work is necessary to achieve a higher accuracy needed for surgical navigation, the results presented in this study are proposed as the first step toward a validated computational framework that could be used for non-invasive localization not only of the DBS system but also brain rhythms triggered by stimulation at both proximal and distal sites in the human central nervous system.

## Introduction

Deep brain stimulation (DBS) of globus pallidus internus (GPi), subthalamic nucleus (STN), and ventral intermediate nucleus (Vim) significantly improves symptoms from patients affected by Parkinson’s disease (PD), essential tremor, dystonia, and obsessive-compulsive disorder that no longer respond to drug therapy. Furthermore, recent evidence suggests that DBS may provide therapeutic benefit to patients with other neurological disorders, including Tourette syndrome, epilepsy, and psychiatric disorders such as depression (Vercueil et al., 2001; Hodaie et al., 2002; Gabriels et al., 2003; Hemm et al., 2005).

Despite the therapeutic success of DBS and its increasing adoption in clinical practice, outcomes are not uniform among different studies (Kleiner Fisman et al., 2006). Significant effort has been dedicated to investigating the wide range of factors that can influence outcomes including stimulation parameters [i.e., contact configurations, frequency, pulse width and voltage (Holsheimer et al., 2000; Moro et al., 2002; O’Suilleabhain et al., 2003; Kuncel and Grill, 2004; McIntyre et al., 2004b; Volkmann et al., 2006)], electrode geometry (Kuncel and Grill, 2004; Butson and McIntyre, 2005; Butson and McIntyre, 2006; Butson et al., 2006), electrode location (Maks et al., 2009), and the electrical properties of the tissues surrounding the implant (Grill and Mortimer, 1994; Grill, 1999; Butson et al., 2007; Yousif et al., 2007). Furthermore, evidence suggests that precise placement of DBS electrodes is key for the optimal clinical outcome of the DBS treatment. A misplaced DBS electrode not only results in decreased effectiveness but could also increase the risk for motor side-effects, such as increased muscular contractions, difficult articulation of speech, oculomotor disturbances or altered sensory phenomena, such as somatosensory paresthesia, diplopia or visual field phosphenes (Montgomery, 2010).

Deep Brain Stimulation is conventionally placed through stereotaxic guidance and microelectrode recording (MER) of single neuron activity. Preoperative images are usually co-registered into the stereotactic coordinate system, and MER is used to confirm the location of the DBS targets by recording and identifying characteristic neuronal discharge patterns that have been associated specifically with GPi, STN, and Vim, as well as other adjacent nuclei. Retrospective analysis of microelectrode track error between the planned trajectory and the microelectrode tip was performed in (Brahimaj et al., 2018), and a total radial error of 1.2 mm was reported. However, MER is time-consuming and requires the patient to be awake due to effects of the anesthesia on neuronal firing. On the other hand, localizing the exact DBS position by visual inspection using conventional imaging techniques such as magnetic resonance imaging (MRI) and computed tomography (CT) during surgery is still a great challenge as they are both affected by metal artifacts (Barrett and Keat, 2004). MRI artifacts induced by DBS have been reported in (Pollo et al., 2004) to be up to 10.4 mm and significant discrepancy between the centers of electrodes estimated by CT and MRI have also been reported. Furthermore, there are also concerns associated with the safety of MRI in patients with DBS electrodes (Gleason et al., 1992; Rezai et al., 2001, 2002, 2004; Bhavaraju et al., 2002).

Several numerical models with varying levels of complexity have been proposed in the literature for low-frequency electromagnetic analysis of the effectiveness of DBS (McIntyre et al., 2004a; Astrom et al., 2009; Grant and Lowery, 2009). Most of these studies model only the electrodes and a few surrounding structures, not the entire human head. Furthermore, available DBS numerical models (McIntyre et al., 2004a, 2007; Astrom et al., 2009; Grant and Lowery, 2009; Miocinovic et al., 2009; Vasques et al., 2009; Yousif and Liu, 2009) are limited by two sequential challenges: 1) prediction of stimulation-induced electromagnetic (EM) field and potential (“forward problem”), and 2) detection/interpretation of EM fields noninvasively from outside the skull (“inverse problem”). We propose a model aimed to bridge the pathway from DBS to noninvasive EEG readout.

To address the first point, we have built an MRI-based anatomical model of the human head previously proposed for RF dosimetry studies (Makris et al., 2008), which has also been adopted for studies with DBS implants in MRI (Angelone et al., 2010; Iacono et al., 2013). We have performed whole-head bioelectromagnetic simulations based on Finite Differences Time Domain (FDTD) method and predicted the DBS signal propagation throughout the head and on the scalp (simulated DBS voltage artifact).

To address the second point, we predicted potential on the scalp to solve the inverse problem and localize the source of the stimulation, i.e., the dipole that generates the DBS stimulation and the large artifact on the EEG. Filtering is commonly used to remove this artifact while preserving the spectral and temporal fidelity of the underlying brain signal. In our methodology, however, we propose to exploit such an artifact present on the EEG recordings of DBS patients and noninvasively “decode” its source with the aim of locating or guiding the DBS electrode implantation during DBS surgery. Dipole source localization – commonly performed to localize the source of brain electrical activity, such as the epileptogenic foci – is proposed in this case to localize the device. In this proof of concept study, we have addressed the technical challenges to achieving a robust DBS localization that could be used in the future for electrode navigation guidance during surgery or spatial localization of stimulus evoked electrical potential to better understand stimulation dose, spatial propagation, or time-dependent effects on distal components in the central nervous system motor network.

## Materials and Methods

### Electromagnetic Simulations

The simulations were based on a head model described in (Makris et al., 2008), based on 1 mm^3^ resolution T1-weighted MRI of a healthy adult human subject. 28 non-brain and 21 brain structural entities were distinguished and segmented on the dataset. Each anatomical structure was converted into its corresponding electrical structural entity as described in (Makris et al., 2008). The result was a heterogeneous model with uniform electrical properties within each anatomical structure. Since the electrical properties of human tissues are frequency-dependent, each electrical structural entity was modeled using the one-pole Debye approximation (Gabriel et al., 1996).

**FIGURE 1:**
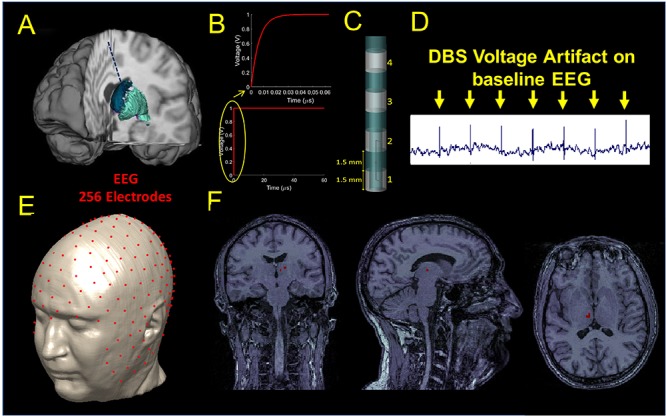
**(A)** Illustration of the DBS placed in the STN. **(B)** waveform used as input for stimulation. **(C)** DBS electrode geometry. **(D)** DBS voltage artifact present on the clinical EEG recordings extracted from Figure 3. **(E)** Source localization of the DBS based on the 256 scalp potential samples. **(F)** Estimated source location on the coronal (let), sagittal (middle), and axial (right) original MRIs.

One left unilateral DBS implant was modeled for the study. The lead was placed along a unique sagittal plane in the subcutaneous structure between the epidermis and the outer table, and then in a coronal plane through the outer table along the brain down to the basal ganglia (Figure 1A). The proximal end of the lead was placed in the neck of the head model and the distal end placed in the white matter region below the thalamus where the subthalamic nucleus is located. The implant was modeled as an insulated lead with an array of four perfect electric conductor cylindrical electrode contacts (Elwassif et al., 2012) at the distal end of the lead (Figure 1C). The length of each electrode was 1.5 mm. A bipolar configuration was considered for the stimulation, and the two electrodes were modeled as a cathode and anode and connected by a conducting wire, as shown in Figure 1C.

A smoothed voltage waveform, resembling the anodic pulse from an implantable pulse generator (IPG) from our clinical data, with an equivalent amplitude of 1 V and 60 μs pulse width was used for stimulation through the DBS electrode. The smoothed voltage step was computed by filtering the 60 μs pulse with a Butterworth low-pass filter of the first order and with a cut-off frequency of 100 MHz (Figure 1B).

Electromagnetic simulations were performed using commercially available software (XFDTD, Remcom, Inc., State College, PA) and each of the electric fields generated by the DBS for three bipolar configurations (1-2, 1-3, 1-4) was transferred into Multiphysics (COMSOL, Burlington MA) for post-processing to calculate the electric potential distribution on the scalp and generate a simulated signal mimicking the magnitude of the DBS voltage artifact present on the EEG recordings of patients with DBS. An example of such an artifact can be seen in Figure 1D and (Frysinger et al., 2006). We called this signal “simulated DBS voltage artifact,” and we used it as input to solve the inverse EEG problem to localize the electric dipole generated by two active DBS electrodes. The three bipolar configurations were chosen as they matched those used in the clinical setting and they produced fields that ranged from narrow (1-2) to wide (1-4) stimulation. Each simulation took 10 days on a workstation that used four NVIDIA Tesla Dual GPU Kepler K80 Graphics Cards with 24 GB of memory each, installed on a 14-core system with 768 GB of RAM. The remaining possible configurations (2-3, 3-4, and 2-4) were not analyzed because they were expected to generate similar results with a shift of 3 mm (1.5 mm length of the electrode + 1.5 mm length of the insulation in between).

The electric scalar potential V was calculated by solving Gauss’s law: -∇⋅(ε_z_ ∇V) = ∇⋅(ε_z_ **E**), where V is the unknown electric potential, **E** is the electric field computed by XFDTD, and ε_z_ is the complex permittivity of tissues. A ground boundary condition (*V* = 0) was set on the side underneath the neck of the bounding box, which encloses the entire head geometrical model. On the remaining sides of the bounding box, an electric insulation boundary condition was used: ε_z_ **n**⋅**E** = 0, where **n** is a vector perpendicular to the bounding box.

### Source Localization

Source localization was performed with Brainstorm (Tadel et al., 2011) in MATLAB (Mathworks, Natick, MA, United States). The original MRI data used to build the numerical head model (Makris et al., 2008) was used to build a three-shell forward head model including scalp, skull, and brain for localization. Once the forward model was built, a 256 channels EEG electrodes net was co-registered onto the head model (Figure 1E). The simulated potentials were then sampled at the 256 channels electrodes positions of the EEG net and imported into Brainstorm. The built-in source localization module of Brainstorm was then used to solve the inverse problem using the unconstrained dynamic Statistical Parametric Maps (dSPM) method with the following default parameters: depth weighting order of 0.5, regularization noise covariance of 0.1 and SNR of 3. Once the inverse problem was solved, full results were exported into MATLAB to find the center of mass of the largest dipole source(s) and its location (Figure 1F). The estimated source location (Sloc) for the three bipolar configurations (Sloc 1-2, Sloc 1-3, and Sloc 1-4, respectively) were compared with the physical center of mass (Mc) of the three pairs of electrodes (Mc 1-2, Mc 1-3, and Mc 1-4, respectively) and the localization error was calculated as the Euclidean distance between the estimated location (Sloc) and the physical one (Mc).

### Clinical Data

All clinical data were acquired according to the IRB (Institutional Review Board) for the protection of human subjects and consist of a cross-sectional sample of resting 10-20 clinical EEG of five PD patients with chronically (>6 weeks) implanted DBS electrodes in the STN. Standard EEG was acquired during delivery of biphasic and bipolar DBS stimulation pulses (amplitude of 3.5 V or 4.5V, width of 60 μs, 10 pulses per second, and two adjacent contacts activated). To assess the accuracy of the FDTD model, the results of the simulation with adjacent contact bipolar activation (i.e., 1–2) were compared with the electric artifact measured in this population. The EEG data were reformatted using a common average reference, linearly scaled to adjust them to the same voltage input (1 V) of the simulated data, and filtered using a high-pass filter with a cut-off frequency of 300 Hz to extract the electrical artifact.

## Results

**FIGURE 2:**
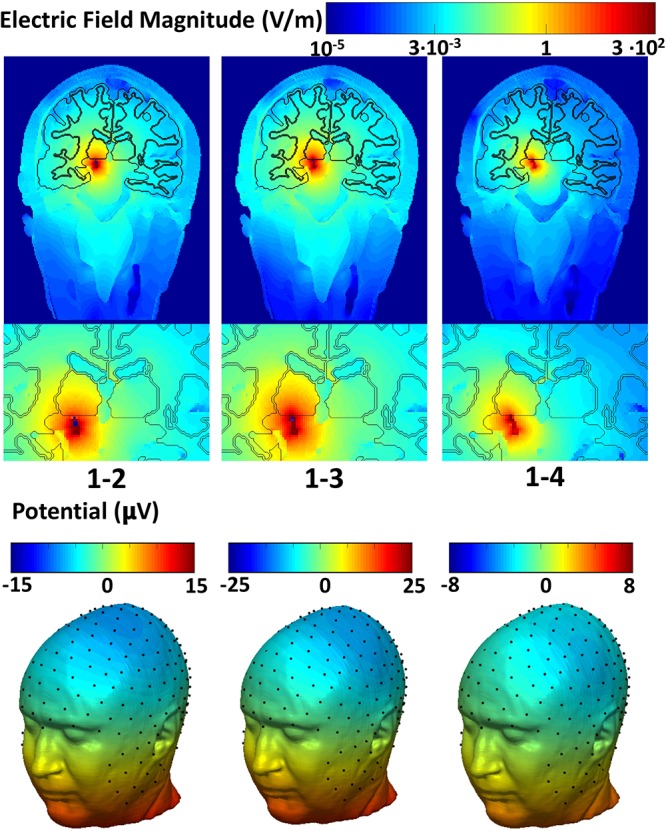
Global and zoomed local spatial distribution of the electric field magnitude overlaid with the precise anatomy of the area surrounding the DBS implant (top row) and the electric potential on the scalp (bottom row).

Figure 2 shows the spatial distribution of the electric field amplitude (top) overlaid with the precise anatomy of the area surrounding the DBS implant and the potential (bottom) on the scalp. The maximum intensity of the electric field produced for the narrow (1-2) and the wide bipolar stimulation configurations (1-3) was 713 V/m and 993 V/m, respectively. The electric field increased up to twofold (1472 V/m) when the widest bipolar configuration (1-4) was used. The peak of the potential was found in proximity to the DBS electrodes and was 5.3 mV, -6 mV, -7.4 mV for the pair 1–2, 1–3, and 1–4, respectively.

The electric potential on the scalp followed a dipole pattern oriented according to the DBS electrodes axis in the head. The 256 sampled scalp potentials (Figure 1E), allowed localization of the DBS electrode pair center of mass (Table 1) with an error of 1.5 cm, 1.4 cm, and 1.2 cm for the three cases, respectively.

**Table 1:** The estimated source location (Sloc) for the three bipolar configurations (Sloc 1-2, Sloc 1-3, and Sloc 1-4, respectively) compared with the corresponding physical centers of mass (Mc) of the same three pairs of electrodes (Mc 1-2, Mc 1-3, and Mc 1-4, respectively).

	Mc 1-2	Sloc 1-2	Mc 1-3	Sloc 1-3	Mc 1-4	Sloc 1-4
x (mm)	111.5	124.3	111	123.5	110.5	121.7
y (mm)	121.5	125.6	122	124.6	122.5	125.5
z (mm)	150.5	157.1	152	156.6	153.5	156.9


**FIGURE 3:**
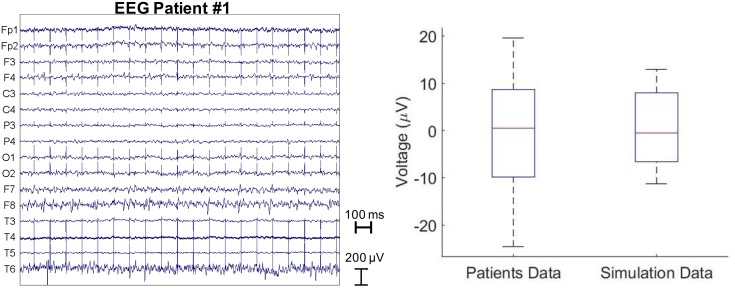
Raw EEG traces of a patient stimulated with 10 Hz and 60 μs DBS pulses (left). The average electric artifact amplitudes over 16 EEG electrodes estimated from five patients vs. those predicted by the electromagnetic simulation with bipolar electrode configuration 1-2 (right).

Furthermore, we compared the results obtained with the FDTD model to a cross-sectional sample of clinical EEG of PD patients with DBS. The amplitude of electrical artifact measured from the EEG clinical data averaged over all the patients and the EEG electrodes was 0 ± 6.6 μV. All EEG potentials are zero mean averaged as a common average reference was used. Figure 3 (left) shows the raw EEG traces of a patient with the DBS on at 10Hz. The average electric artifact amplitudes over 16 EEG electrodes estimated from five patients were compared with those predicted by the electromagnetic simulation with bipolar electrode configuration 1-2 (right).

## Discussion

Intraoperative brain imaging would be the optimal approach for guiding DBS surgery. However, one of the main concerns regarding the use of imaging systems such as MRI for DBS patients is related to possible induced heating. There is an example of a patient reporting edema near the tip of one of the electrodes with the consequent paralysis after undergoing MRI (Henderson et al., 2005) (notably, the FDA-approved manufacturer’s guidelines were not followed). Additionally, MRI acquisition considerably lengthens the duration of the surgery and requires the use of general anesthesia for targeting, without the ability to adjust the electrode position in real time based on MER and/or assessment of stimulation effectiveness and side effects during surgery. EEG has the potential for being a high impact and disruptive technology compared to the intra-operative imaging for non-invasive guidance of DBS surgery procedures because of the low cost of the device, installation, operation, ease of use and safety. When performing EEG on a patient with an active DBS, the DBS pulse typically constitutes an artifact on the EEG signal. In this paper, we have instead studied such pulse and shown that it is possible to non-invasively localize a DBS electrode analyzing the distribution of electric potential on the scalp generated by this DBS pulse.

Notably, the method is still in its infancy and significant limitations still exist with EEG, most important of which is the accuracy of the localization of the brain sources from the recorded EEG due to the ill-posed nature of the methodology which leads to multiple solutions (Bonmassar, 2016). An error of 1-3 cm has been reported by studies investigating source localization using simplified spherical models (Acar and Makeig, 2013). In line with these studies, we report a maximum localization error of 1.5 cm.

In this study we describe a set of technical strategies that can be adapted to improve localization accuracy further. To the best of our knowledge, the proposed model is the first of its kind and is provided as a proof-of-concept methodology for device localization. Further methods are under development for minimizing/eliminating the stimulus artifact from electrophysiological recordings (Walker et al., 2012a,b). Future studies could conceivably expand on these methods, in order to better understand how DBS interacts with local and distant neuronal elements as a function of time after the stimulus pulse. For these explorations, confirmation of the known location of the DBS electrode with the stimulus transient could be used to constrain other investigations of brain activation as a function of time after the stimulus pulse.

These methods have some potential limitations. The most critical source of error is the forward head model employed in the source localization algorithm. Herein we used a forward head model that was automatically segmented into three tissue types: skin, skull, and brain. Errors due to automatic segmentation can jeopardize the localization accuracy. Furthermore, taking into account the anisotropic conductivity of tissues can improve volume conduction modeling.

Additionally, uncertainties in electrical parameters should be taken into account as a dominant source of localization error in the simulation results. For example, EEG models are sensitive to the skull conductivity and anisotropy. In addition, electrical properties may vary between individuals (Atefi, 2015; Atefi et al., 2016).

Another possible source of errors is due to the co-registration of the EEG cap onto the head model. Co-registration was performed by visually adjusting the position of the electrodes on the scalp of the virtual patient. More accurate co-registration strategies, e.g., non-linear co-registration methods, could be performed to fit the EEG electrode cap on the head.

The performance of the proposed source localization method should be assessed in the presence of noise (i.e., which is in our case better than standard EEG given that the DBS artifact is usually greater than any physiological EEG signal), using reduced electrode numbers (16, 32, 64, and 128 electrodes) and different localization algorithms such as the Minimum Norm Estimate (MNE) and LORETA (Pascual-Marqui, 1999). A new type of source localization, namely Direct Electromagnetic Source Tomographic Imaging Neurotechnology (DESTIN), may allow us to study DBS patients during DBS surgery not using a traditional source localization approach but rather a time of flight localization as it is similarly done in PET (Bonmassar, 2016). This could result in improved results as well as in decreased computational load.

Additional error mitigation – independent from the source localization method – could be achieved by improving the prediction of the simulated EEG potential on the scalp used to feed the inverse problem. A uniform 1 mm^3^ electric grid was used to discretize the head and the DBS model in the FDTD EM simulations due to available computational resources. However, a multi-scale discretization with both millimetric and micrometric resolution, as used in (Iacono et al., 2013) may be needed to calculate a more accurate solution of the electric field generated by the DBS. Micro-resolution is crucial in order to precisely sample objects like DBS electrodes and to avoid errors such as staircasing (Railton and Schneider, 1999; Gajsek et al., 2002). The millimetric resolution is also crucial because performing simulations using a uniform submillimetric resolution for the entire head (Iacono et al., 2015) would require an extremely long processing time with the available computational resources. The uniform milli-resolution modeling alone – that was used in our simulations – may have resulted in a loss of accuracy in the mimicked scalp electric potential which in turn can confound the source localization.

Finally, the electrical properties of the head model used in the FDTD simulations were considered isotropic (i.e., the Debye model is isotropic). The inclusion of anisotropic electrical properties may enhance the accuracy of the simulated electric potential on the scalp. However, due to limitations in memory of the GPU cards, the inclusion of the anisotropic material was not feasible. Furthermore, the dielectric properties of the electrode/tissue interface did not include a capacitive component to model the drop in voltage that occurs in the transition from the polarization of the DBS electrode contact to the ionic medium because of convergence issues with the FDTD algorithm (Yousif and Liu, 2007).

Nevertheless, an improved EEG localization method tailored specifically to DBS, like the one proposed in this paper, could one day revolutionize DBS implantation resulting in a more uniform procedure across centers, using the EEG as a non-invasive image-guided tool. Pre-operative MRI data of the patients could be segmented in advance to generate the forward model. Real-time EEG recording with the implantable pulse generator of the DBS turned ON could be filtered to isolate the DBS artifact (Allen et al., 2010) and used to localize the electrode in the brain during surgical navigation similarly to how a Global Positioning System (GPS) is used in terrestrial navigation. Sterilization of the EEG system could be one obstacle to put in practice such a procedure while performing a stereotaxic surgery. However, safe use of disposable sterilized high-density EEG net has been previously reported (Yamazaki et al., 2013; Ahmadi et al., 2016).

Other applications may stem from this methodology: for example, the DBS artifact present on EEG recording can be used during the post-operative reprogramming of the IPG and provide the clinician with information about the composition and electrical changes of the tissues that surround the electrode, which may be important in patients with reduced stimulation efficacy to establish whether glial scar or changes in electrode impedance may play a role in changing clinical state after surgery. Furthermore, closed-loop smart DBS devices have already been proposed to dynamically and automatically adjust the stimulation to suppress pathological synchronization in patients with PD (Eusebio et al., 2011; Rosin et al., 2011). In these devices, the EEG electrical artifact may represent a simple yet widely available means of obtaining DBS pulse amplitude information in order to adjust the stimulation automatically during IPG programming/calibration. Finally, automatic calibration based on EEG artifact may become even more significant when applied to psychiatric disorders like obsessive-compulsive disorder and depression where the symptoms and the effects of the therapy are more difficult to observe and quantify.

## Conclusion

We presented a computational modeling framework proposed as a proof-of-concept for non-invasive localization of DBS by means of EEG recording on the scalp. Numerical results were comparable with EEG clinical data recorded from PD patients with implanted DBS. Our findings showed that the subcortical DBS sources were localized using EEG data on the scalp with a ∼1 cm accuracy. While we acknowledge that further work is necessary to achieve a higher accuracy needed for surgical navigation, the results presented in this study are proposed as the first step toward a validated computational framework that could be used for non-invasive localization not only of the DBS system but also for other types of medical implants.

## Author Contributions

GB conceived the project. MII, SRA, and GB designed the study, performed the numerical modeling, and analyzed the data. HCW acquired the data. LMA, HCW, and LM provided scientific feedback. MII wrote the manuscript. All authors reviewed the manuscript.

## Disclaimer

The mention of commercial products, their sources, or their use in connection with material reported herein is not to be construed as either an actual or implied endorsement of such products by the Department of Health and Human Services.

## Conflict of Interest Statement

HCW receives funding for fellowship training from Medtronic. HCW also serves as a consultant for Medtronic and Boston Scientific. The remaining authors declare that the research was conducted in the absence of any commercial or financial relationships that could be construed as a potential conflict of interest. The handling Editor is currently co-organizing a Research Topic with one of the authors LMA, and confirms the absence of any other collaboration.
